# A Study on Software-based Sensing Technology for Multiple Object Control in AR Video

**DOI:** 10.3390/s101109857

**Published:** 2010-11-02

**Authors:** Sungmo Jung, Jae-gu Song, Dae-Joon Hwang, Jae Young Ahn, Seoksoo Kim

**Affiliations:** 1 Hannam University of Department of Multimedia, Daejeon, Korea; E-Mails: sungmoj@gmail.com (S.J.); bhas9@paran.com (J.-G.S.); 2 Sungkyunkwan University of Information & Communication Engineering, Suwon, Korea; E-Mail: djhwang@skku.edu; 3 ETRI Standards Research Center, Daejeon, Korea; E-Mail: ahnjy@etri.re.kr

**Keywords:** software-based sensing, multiple object detection, multiple object loading, multiple object control, computer vision, augmented reality

## Abstract

Researches on Augmented Reality (AR) have recently received attention. With these, the Machine-to-Machine (M2M) market has started to be active and there are numerous efforts to apply this to real life in all sectors of society. To date, the M2M market has applied the existing marker-based AR technology in entertainment, business and other industries. With the existing marker-based AR technology, a designated object can only be loaded on the screen from one marker and a marker has to be added to load on the screen the same object again. This situation creates a problem where the relevant marker’should be extracted and printed in screen so that loading of the multiple objects is enabled. However, since the distance between markers will not be measured in the process of detecting and copying markers, the markers can be overlapped and thus the objects would not be augmented. To solve this problem, a circle having the longest radius needs to be created from a focal point of a marker to be copied, so that no object is copied within the confines of the circle. In this paper, software-based sensing technology for multiple object detection and loading using PPHT has been developed and overlapping marker control according to multiple object control has been studied using the Bresenham and Mean Shift algorithms.

## 1. Introduction

Augmented Reality (AR) [1] refers to a virtual world which can provide a sense of reality in interactions with users, where images of real world and virtual graphic objects are generated by computer, and it is almost a lower level of virtual reality rather than a virtual reality realizing a complete immersion environment. Core technologies to establish AR systems include marker detection to locate an object, matching, tracking, camera calibration to synthesize virtual objects into actual images and calculate camera variables, display, and 3D modeling technology [2]. That is, it is a technology field that provides the image with information such as characters and graphic objects in real life to enhance users’ understanding of the situation of moving objects in 3D space [3].

Initially, the AR system had been developed by Ivan Sutherland in 1968 using Head Mounted Displays (HMDs) [4], which could only display some very simple wire frames in real time, due to the limitations of the computer performance at the time [5]. After that, in 1992, Tom Caudell developed display technology using HMDs in earnest and began to use the term, AR, and since then, consistent research has been conducted. In modern times, as the performance of easy-to-carry computing terminals and display environments has been developed, AR technology has been rapidly developed, and is being used in many areas is being actively conducted with consistently increasing demands. For the realization of AR is real life, what point of the object image captured by camera should be augmented needs to be decided and augmentation methods are classified into marker recognition methods and markerless recognition methods. Since markerless recognition methods require feature points which are extracted from the image captured by camera, much time and labor are needed for image processing [6]. Besides much research is required to increase extraction rates and the research on this topic are in active progress. Marker recognition methods are a method of augmenting an object in a point where the computer recognizes the tag interface serving as a pointer connecting the real world and the virtual world, which is called a marker. This method, due to its high recognition rates and easy use, is widely used in the realization of AR [7].

The existing marker-based AR technology can only load a designated object in one marker and actually has to add another marker to load the same object again. To solve this problem, the relevant marker should be extracted and printed onscreen so that the loading of multiple objects is enabled. Marker-based AR uses the Progressive Probabilistic Hough Transform (PPHT) [8] for multiple-object loading technology to detect and copy the same marker, and in the case where only this method is used, markers can be overlapped and therefore the object will not be augmented.

In this paper, an AR multiple-object loading technology has been developed. By utilizing the Bresenham algorithm for AR multiple-object loading, an area was designated to prevent markers from overlapping based on a focal point of the marker and marker overlapping control was thus made possible.

## 2. Related Research

### 2.1. Hough Transform

The Hough transform is a method for detecting straight lines, circles or other simple shapes in an mage [9]. The Hough transform is based on the fact that there are countless straight lines passing through any point in the binary image [10].

If a line has a *y*-intercept of b and a slope of a (*y = ax + b*), a point on the (*x, y*) coordinate plane is expressed as a straight line on the (*a, b*) coordinate plane. In the case where no zero pixels in the input image are all expressed as straight lines on the (*a, b*) coordinate plane image and the pixel values located where the lines pass through are accumulated, a line on the (*x, y*) coordinate plane is shown to have a local maximum value on the (*a, b*) coordinate plane. Since all loci of the lines expressed by each point are summed, the (*a, b*) coordinate plane is commonly called an accumulator plane [11].

However, the method of expressing a straight line with a slope and a y-intercept is not appropriate to express all straight lines on the (*x, y*) coordinate plane. This is because to express the common straight lines of which slopes range from − ∞ to + ∞ in the binary image is difficult. Therefore, in practical realization, it is expressed as a point, (*ρ, θ*) on the polar coordinate system by means of another method. A line expressed as (*ρ, θ*) on the polar coordinate system means a straight line perpendicular to a line passing through this point and the origin, which is expressed in the following numerical formula (1):

(1) ρ=xcosθ+ysinθ

**Figure 1.**
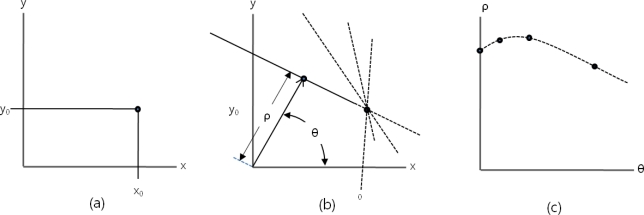
Common accumulator plane expressed on the polar coordinate system. **(a)** A point (*x_0_, y_0_*). **(b)** The image coordinate system becomes a point. **(c)** The polar coordinate system intersects and it can be expressed as a line.

A point (*x_0_, y_0_*) on the image coordinate system, as shown in Figure 1(a), becomes a point in Figure 1(b) where many straight lines expressed as (*ρ, θ*) on the polar coordinate system intersect and it can be expressed as a line in Figure 1(c) on the (*ρ, θ*) plane as follows.

In this paper, reflecting local maximum values on the (*ρ, θ*) coordinate plane, the marker recognition method was expanded to include not only a simple straight line but other general shapes.

### 2.2. Bresenham Algorithm

Most of the various line algorithms require low performance floating point division operations. However, the rasterization which was proposed by Bresenham is a super high speed algorithm that rasterizes straight lines/circles/ellipses using only addition/subtraction operations of pure integers [12]. The fundamental feature of the Bresenham algorithm [13] are the accumulated ‘error terms’. That is, the computer screen corresponds to a great 2-dimensional array of colored squares. An act of drawing a straight line (or another figure) at this point is a process of ‘approximating’ a continuous coordinate system of real numbers into a discontinuous coordinate system. Therefore, a dot usually provides an error between discontinuous coordinate systems printed in the original coordinate system of the line and the screen, and the key concept of Bresenham algorithm is to select a dot which best minimizes this error by drawing the straight lines (or another figures). The process of drawing lines using Bresenham algorithm is as follows:

1. Array *p1* and *p2*, the two dots representing the line in the order of coordinate axes. At this point, if the slope of the line is less than 1, array in increasing order of coordinate *x* and if greater than 1, array in increasing order of coordinate *y*. (Here assumed to be arrayed to increasing order of *x*)
2. Begin with the first dot out of the dots arrayed.
3. Make a dot at the present location.
4. Provide setting the next location. Then, increase the location of the present pixel by one to increasing order of coordinate *x*.
5. Calculate the error value at the next location. Here, the error term is the addition of the differences between *y* coordinate values of *p1* and *p2*.
6. Compare the error terms and examine if the error portion is greater than one pixel. That is, after comparing the error terms up to now and the difference between *x* coordinate values of *p1* and *p2*, increase the coordinate value by one to increasing order of coordinate *y* if the error term is greater than the difference.
7. Repeat (3) to (6) until the last coordinate is dotted.

For drawing of a quadrangle, the process of drawing four lines using Bresenham algorithm is repeated. The process of drawing a circle represented by the equation, *x*^2^ + *y*^2^ = *r*^2^ the fundamental of algorithm to be used in this paper is as follows:

1. Begin with a fixed point on the top of the circle. Here, draw a quarter circle clockwise and repeat this circle four times.
2. Make a dot in the present coordinate.
3. Increase the coordinate by one to increasing order of coordinate *x*.
4. Then decide *y* coordinate. Decide one out of *y* or *y−1* for y coordinate. If *x*^2^ + (*y* − 1)^2^ < *x*^2^ + *y*^2^ < *r*^2^ is valid, y becomes the next coordinate and if *r*^2^ < *x*^2^ + (*y* − 1)^2^ < *x*^2^ + *y*^2^ is valid, *y*−*1* becomes the one. In other cases except for these, the error can be ignored with any dot between the two.
5. Repeat (2) to (4) until *x == y*.
6. Begin with the fixed point on the right side of the circle.
7. Make a dot in the present coordinate.
8. Increase the coordinate by one to increasing order of coordinate *y*.
9. Then decide *x* coordinate. Decide one out of *x* or *x−1* for *x* coordinate. If (*x* − 1)^2^ + y^2^ < *x*^2^ + *y*^2^ < *r*^2^ is valid, *x* becomes the next coordinate and if *r*^2^ < (*x* − 1)^2^ + *y*^2^ < *x*^2^ + *y*^2^ is valid, *x−1* becomes the one. In other cases except for these, the error can be ignored with any dot between the two.
10. Repeat (7) to (9) until *x==y*.

The algorithm of drawing an ellipse used in this paper is similar to the case of a circle, and the algorithm was applied by changing the equation and calculating the point turning over the direction of axis.

### 2.3. Mean Shift Algorithm

The mean shift algorithm [14] is a nonparametric statistical method for detecting a major mode among distributed sample points. The multivariate kernel density estimator under the condition of *R^d^* (*d* dimensional space) and *{x_i_}_i=1…n_* (*n* number of data set) is as follows:

(2) $$\hat{f}(x)=\frac{1}{{\mathrm{nh}}^{d}}\sum_{i=1}^{n}K\left(\frac{x-x_{i}}{h}\right)$$

here, *K*(*x*) refers to the kernel and h, a radius of a window. The mode that density *f*(*x*) becomes a local maximum is located where gradient *f*(*x*) is 0. This is expressed as follows:

(3) g(x)=−k′(x)

here, *k*(*x*) is a profile of kernel *K*(*x*). If *g*(*x*) is a profile of kernel *G*(*x*), *G* is called a shadow of kernel *K* [15]. Detecting a density mode means estimating the density to find a convergent point of the mean shift. Thus, kernel *G* is used in order to find out a convergent point where the difference between *x* and a sample mean is 0. The following formula shows this:

(4) $$m_{h.G(x)}=\frac{\sum_{i=1}^{n}x_{i}g({\left\|\frac{x-x_{i}}{h}\right\|}^{2})}{\sum_{i=1}^{n}g({\left\|\frac{x-x_{i}}{h}\right\|}^{2})}-x$$

Mean shift m_h,G(x)_ is proportional to a gradient of normalized density and always changes in the direction that a density function shows the greatest increase.

Formula (4) can be expressed as Formula (5) by using a shadow kernel *G* to express a series of locations of the sample means as *{y_j_}_j_* _= 1, 2, …,_:

(5) $$y_{j+1}=\frac{\sum_{i=1}^{n}x_{i}g({\left\|\frac{x-x_{i}}{h}\right\|}^{2})}{\sum_{i=1}^{n}g({\left\|\frac{x-x_{i}}{h}\right\|}^{2})}$$

here, *y_j+1_* refers to the weighted mean at *y_i_*, calculated by kernel *G*, and *y_1_*, initial center of kernel position. The mean shift algorithm repeats the following process so as to find out the potential centers of the cluster [16]:

1. Calculation of mean shift vector *m_h,g(x)_*
2. Renewal of *y_j_*, position of the current anchor point

In this paper, based on the concept of the mean shift algorithm as well as “motion segmentation and analysis in video sequences [17]” and “a graph-segment-based unsupervised classification for multispectral remote sensing images [18]”, numerous micro-domains such as noises are removed from a final outcome image by effectively combining image domains, taking into account that objects should be augmented by markers when loading/controlling multiple objects. The study also suggests that the size of a domain be limited for minimum merging of adjacent domains.

## 3. Software-based Sensing Technology for Multiple Objects Detection and Loading

### 3.1. Maker Detection Using PPHT

In this paper, an 8-bit input image is processed into a binary image, no zero pixels are handled all equally and an N*1 sized matrix is stored in the memory storage as a pointer of the space where the result of Hough transform is to be stored. The most important thing in case of detecting a marker using PPHT, is to set a critical value to estimate it as a straight line on the accumulator plane. This paper set the critical value to optimize this in experimental condition and derived the resulting image.

**Figure 2.**
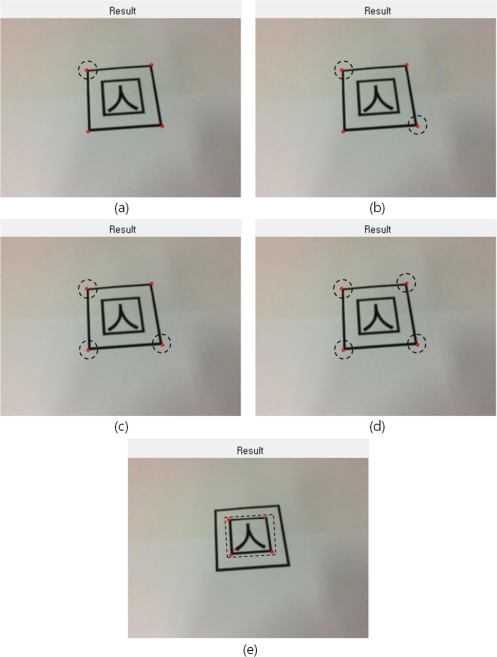
Edge points and detection of tag ROI. **(a)** Selected and an edge point locating farthest from the point is extracted by means of Pythagoras’ theorem. **(b)** An edge point lying farthest from the extracted point is investigated. **(c)** An edge point locating farthest from the obtained edge point is extracted again. **(d)** A point which maximizes the area of the square becomes the rest one point. **(e)** The marker in marker tag can be extracted with edge points.

The methods of implementing the edge tracing algorithm to achieve contour information of the marker’s contour out of the result image derived, and extracting edge points from the contour information existing within the marker region are the following: first, if all edge points inside or outside of the marker region appear, one point out of tag region’s contour information is selected and an edge point locating farthest from the point is extracted by means of Pythagoras’ theorem as shown in Figure 2(a). Next, an edge point lying farthest from the extracted point is investigated as in Figure 2(b), and then an edge point locating farthest from the obtained edge point is extracted again as in Figure 2(c). As illustrated in Figure 2(d), a point which maximizes the area of the square becomes the rest one point. Based on the four points extracted, the marker ROI (region of interest) in marker tag can be extracted as shown in Figure 2(e):

(6) $$\begin{array}{ll} \mathrm{RectArea} & \\ & =\frac{1}{2}(x_{1}y_{2}-x_{2}y_{1}+x_{2}y_{3}-x_{3}y_{2}+\cdots +x_{n}y_{1}-x_{1}y_{n})\\ & =\frac{1}{2}x_{1}(y_{2}-y_{n})+x_{2}(y_{3}-y_{1})+\cdots +x_{n}(y_{1}-y_{n-1}) \end{array}$$

PPHT (Progressive Probabilistic Hough Transform) [19] which is to be finally used for marker detection can calculate the beginning and the end of each line as well as the direction of line. This method only considers some voluntarily selected edge pixels, not increasing values for all edge pixels on the accumulator plane.

**Figure 3.**
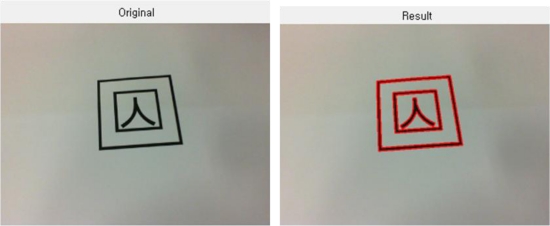
Marker detection using PPHT.

In case a value in a specific location appears to be higher than the critical value, no more value increase is allowed, and the edge pixels displayed by this point into a straight line will be ignored in further calculations. Besides, since this method can reduce the calculation time drastically, it is highly effective in real time marker detection, as shown in Figure 3.

The above figures shows the tag detected using PPHT, from the original image captured from Webcam. For easy distinction, the detected parts are marked in bold.

### 3.2. Video Output of Replicated Marker

This research suggests a template matching method that makes warping into the marker registered in a square form from edge points which are extracted within the image, and performs matching. First of all, when the four edge points outside the marker are designated as (*x_1_*, *y_1_*), (*x_2_*, *y_2_*), (*x_3_*, *y_3_*), (*x_4_*, *y_4_*) respectively, a point where the straight line passing through (*x_1_*, *y_1_*) and (*x_3_*, *y_3_*), and the line through (*x_2_, y_2_*) and (*x_4_, y_4_*) intersect becomes the center of the marker. The formula to calculate the center of the marker using the equation of straight line is as below:

(7) $$C_{y}=\left(\frac{y_{3}-y_{1}}{x_{3}-x_{1}}\right)\left(C_{x}-x_{1}\right)+y_{1}$$

(8) $$C_{y}=\left(\frac{y_{4}-y_{2}}{x_{4}-x_{2}}\right)\left(C_{x}-x_{2}\right)+y_{2}$$

*C_x_* is obtained by calculating formulas (7–8) and then *C_y_* is obtained by substituting this for formula (7). If the height of the tag of a specific standard and the height of the extracted marker tag set to *h_r_* and *h_d_* respectively, the *Sdiff (Scale difference ratio)* according to the change of plane for the extraction can be calculated as the following formula (9):

(9) $$S_{\mathrm{diff}}=\frac{h_{d}}{h_{r}}\times 100$$

If the center of the marker which is finally calculated is estimated as the origin, the size error correction (*T_total_*) can be expressed as the formula (10), and *D_t_* here refers to transform matrix:

(10) $$T_{\mathrm{total}}=\left[\begin{array}{ccc} \frac{1}{S_{\mathrm{diff}}} & 0 & 0\\ 0 & \frac{1}{S_{\mathrm{diff}}} & 0\\ 0 & 0 & 1 \end{array}\right]\times D_{T}$$

**Figure 4.**
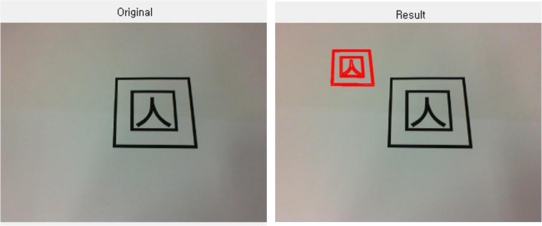
Copying and moving of the detected marker.

Figure 4 below shows that a marker tag extracted from the image is copied and moved to the desired location.

The binarized image of the marker marked for the function of a marker is shown below.

## 4. Deduction of Markers Overlapping Problem

**Figure 6.**
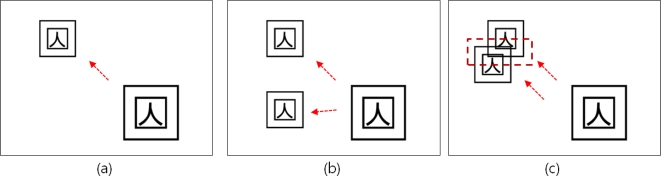
Deduction of markers overlapping problem.

This paper is based on the assumption that an 8-bit input image is processed in binary image data, non-zero pixels are all treated as the same and N*1 matrix is stored in memory storage for the pointer of a space where copied markers will be stored. Figure 6(a) shows the process of detecting the marker using PPHT and copying one marker. Two markers are copied through multi markers copying as shown in Figure 6(b). In this way, n markers can be copied and therefore objects can be augmented, but if markers are overlapped as in Figure 6(c), the object cannot be augmented.

Therefore in this paper, to control the overlapping markers problem, the marker area was set up with Bresenham algorithm applied and the problem was solved by designating non-overlapping area based on the marker’s focal point.

## 5. How to Create Marker Area by Applying

### 5.1. Calculation of the Marker’S Focal Point through Detection of Edge Points

**Figure 7.**
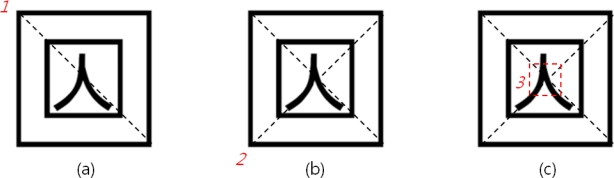
Extraction of a marker’s focal point.

For the calculation of the marker’s focal point, the contour information of the marker area in the image was acquired by conducting the edge tracing algorithm and the edge points were extracted from the contour information inside the marker area. The process of extracting the marker’s focal point with the above formula is as indicated in Figure 7.

### 5.2. Creation of Non-Overlapping Area, Based on Marker’S Focal Point

When calculating a circle’s area from the marker’s focal point in a square, the area should be divided every 45 degrees. However, the marker extracted from the image is not recognized as a 100% circle, an ellipse’s area needs to be considered. Since an ellipse cannot be calculated or divided into every 45 degree, when considering a line drawn at a point with a gentle slope, the slope $\frac{d_{y}}{d_{x}}$ should be regarded as 1 or −1.

In the elliptic equation, $\frac{x^{2}}{a^{2}}+\frac{y^{2}}{b^{2}}=1$, a point with the slope of −1 is $x=\frac{a^{2}}{\mathrm{sqrt}(a^{2}+b^{2})}$, $y=\frac{b^{2}}{\mathrm{sqrt}(a^{2}+b^{2})}$.

Therefore, *x* should be an independent variable to this point and *y* should be one for the remaining part. Other quadrants are drawn using its symmetry properties. If the point is (*x, y*), the next point possible is (*x + 1, y*) or (*x + 1, y−1*), and it is determined by seeing whether the middle point of these points is fallen inside or outside of the ellipse.

**Figure 8.**
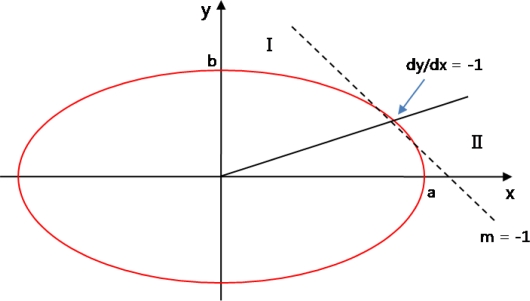
Basic structure to create an ellipse.

Figure 8 describes the process of creating an ellipse with the discriminant, $d=F(x+1,\frac{y-1}{2})$, for *F*(*x, y*) = *b*^2^ · *x*^2^ + *a*^2^ · *y*^2^ − *a*^2^ · *b*^2^.

If *d* value is less than 0, (*x+1, y*) and greater than 0, (*x+1, y−1*) is selected. For the update of the discriminant to be calculated on the marker’s focal point coordinate, if *d_old* is less than 0, it is *d_new = F*(*x + 2, y*) *= d_old + b*^2^ · (2 · (*x* + 1) + 1 and if *d_old* is greater than 0, it is $d\_\mathrm{new}=F(x+2,\frac{y-3}{2})=d\_\mathrm{old}+|b^{2}\cdot (2\cdot (x+1)+1)-a^{2}(2\cdot (y-1).)$

**Figure 9.**
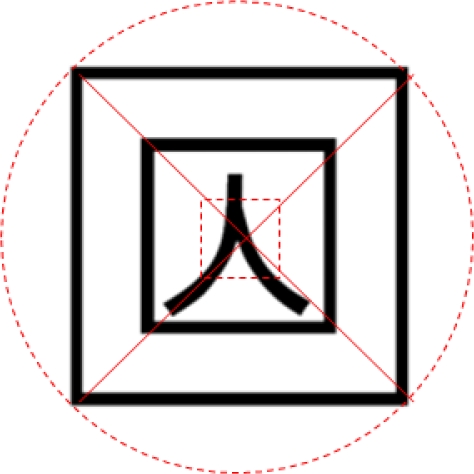
Creation of marker’s focal point-based area.

As the independent variables of *x* are between 0 and −1 of the slope, for an interval of independent variables in *y*, the roles are just changed in the numerical formulas calculating independent variables in *x*. Figure 9 shows the process of creating an ellipse’s area based on the marker’s focal point.

**Figure 10.**
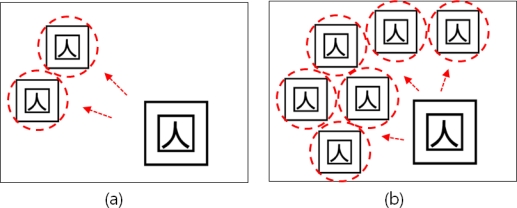
Overlapping control during marker creation, by designating the created areas as non-overlapping areas.

In case two markers are copied after the area created is designated as non-overlapping area, the applicable area is not interfered as in Figure 10(a). Besides, if more than two markers are to be copied, as the number of markers printed in one screen is automatically limited, the markers overlapping problem can be solved and the number of copying operations can be regulated according to the scale of the marker.

### 5.3. Removal of Micro-Domains Using the Mean Shift Algorithm

Image segmentation using the mean shift algorithm can produce the best result when the sizes of a space window and a keypoint window are properly selected. However, if the sizes of the two windows are small, there can be excessive micro-domains, resulting in excessive segmentation also. In order to overcome such a problem, this study suggests a method whereby domains in an excessively segmented image are merged using critical values and boundaries of merged domains are preserved. The processing of an input image is largely divided into two steps: segmentation by the mean shift algorithm and merger of the outcome images. The first step, segmentation by the mean shift algorithm, is as follows:

1. Convert an RGB color domain of an input image into a CIE LUV color domain
2. Map each pixel of the converted image by a joint domain of a spatial-range domain
3. Classify the mapped pixels into clusters. Classify pixels within a window in a joint domain into a single cluster of those with a similar keypoint.
4. Calculate a mean of a cluster
5. Calculate the value of the mean shift
6. Find the convergent point. If can’t, move the center of a window to the mean calculated by (4) and repeat (3∼6). End the segmentation process if the convergent point was found.

Image segmentation using the mean shift algorithm could be done excessively according to sizes of a window. The suggested method converts an RGB color domain into an HIS [20] color domain for merging domains in an excessively segmented image. This method is frequently used for color segmentation, for it is less affected by non-uniform illumination such as shadow, projection, and reflected light. However, if domains are merged by colors, objects of different adjacent domains but of the same color could be merged, which could obscure boundaries of major domains. In addition, most domains with low brightness are those of shadow and domains of low chroma are dim areas. Therefore, there are colour values not perceived in a dark or dim areas, which may cause inaccurate merger of domains.

This research, therefore, applies limiters for the merger of domains to solve the problem. By applying limits to features existing in the domain of the boundaries, the boundaries are preserved and influences of low brightness and chromas are minimized. Such limits include chroma, brightness, and an edge of an HIS color domain. The edge is calculated by applying the Sobel operation to the brightness of an HIS color domain. If brightness and chroma of a pixel to be processed is lower than that of a critical value, respectively, or if the edge is higher than a critical value, the pixel is not merged.

According to the suggested method, limiting segments of boundaries are applied to a pixel to be processed in the initial labeled domain prior to merger of domains. If any restrictive condition is applicable to a pixel, that pixel is not treated for merger. Also, if no restrictive condition is applicable, adjacent domains of the same color are merged. The following formula shows a restrictive condition (*FR*) while *P_i_, P_s_*, and *P_e_* refers to brightness, chroma, and edge of a pixel to be processed and *Th_i_, Th_s_,* and *Th_e_*, critical value of the brightness, chroma, and edge:

(11) FR={not process, Pi<Thi or Ps<Ths or P2>Themerging process, otherwise

After merger of domains, there are many micro-domains such as a noise in the resulting image. This study suggests that the area of a domain be limited. Using the histogram method, frequency of a domain label is examined and a domain smaller than a critical value is merged with others through Euclidean distance. That is, domains are merged so that chrominance distance is minimized. The following formula shows the Euclidean distance when the color of pixel *c1* and *c2* is referred to as (*R_1_*, *G_1_*, *B_1_*) and (*R*_2_, *G*_2_, *B*_2_):

(12) $$d_{E}(c_{1},c_{2})=\sqrt{{\left(R_{1}-R_{2}\right)}^{2}+{\left(G_{1}-G_{2}\right)}^{2}+{\left(B_{1}-B_{2}\right)}^{2}}$$

The following figure is the final outcome.

## 6. Conclusions

In this paper, software-based sensing technology for multiple objects detection and loading using PPHT has been developed. Marker overlap control according to multiple objects control has been studied using the Bresenham and Mean Shift algorithms.

The main contribution of this paper are to solve the problem of the existing marker-based AR technology, one marker to one object loading, PPHT-based AR multiple objects loading technology which enables multiple objects loading without another marker added has been developed, by extracting the same marker from the image and copying it at a desirable location. The overlapping marker problem was deduced in PPHT-based AR multiple objects loading technology which realized one marker N objects loading. To solve the problem, the method of setting a marker area using the Bresenham and Mean Shift algorithms was researched, and methods of calculating the marker’s focal point through detection of edge points and of creating non-overlapping area based on the marker’s focal point were suggested.

This research has the advantage that it can solve the problems that may occur during marker copying and limit the number of markers printed in one screen automatically. This research can be most widely used since it is applicable to research on feature point extraction of not only marker-based AR but also markerless-based AR. However, multiple object loading technology has the limitation that only marker-based AR can be used.

Based on this research, an application that enables real objects loading will be added to the future research. Besides, research on an algorithm which reduces more the marker detection time and a system that implements marker detection smoothly even in any environment must to be carried out. A plan to increase or decrease the number of markers printed in one screen should be prepared through the development of a technology of regulating the scale during marker copying. Because of the real time nature of AR, of the development of very fast and correct algorithms is required.

## Figures and Tables

**Figure 5.**
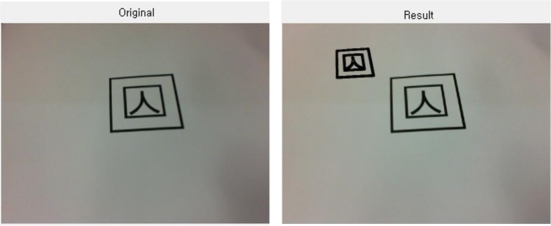
Binarization of the copied and moved marker.

**Figure 11.**
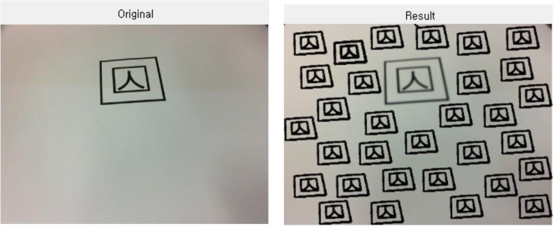
Multiple objects loading after removing micro-domains.
